# Supplementary material for: Incomplete transcripts dominate the Mycobacterium tuberculosis transcriptome
Source: Nature. 2024 Feb 28;627(8003):424–30. doi: 10.1038/s41586-024-07105-9 (PMC10937400; doi:10.1038/s41586-024-07105-9)
Supplement: Supplementary file 1 — Supplementary Fig. 1 (original gels) and full descriptions for Supplementary Tables 1–4. [file 41586_2024_7105_MOESM1_ESM.pdf]

nature portfolio

https://doi.org/10.1038/s41586-024-07105-9

Supplementary information

Incomplete transcripts dominate the *Mycobacterium tuberculosis* transcriptome

In the format provided by the authors and unedited

Incomplete transcripts dominate the *Mycobacterium tuberculosis* transcriptome

Xiangwu Ju, Shuqi Li, Ruby Froom, Ling Wang, Mirjana Lilic, Madeleine Delbeau, Elizabeth A. Campbell, Jeremy M. Rock*, Shixin Liu*

Corresponding authors: Shixin Liu, Jeremy Rock

Supplementary Information

Supplementary Figure 1:Original images of gel electrophoresis results.

Extended Data Fig. 1b

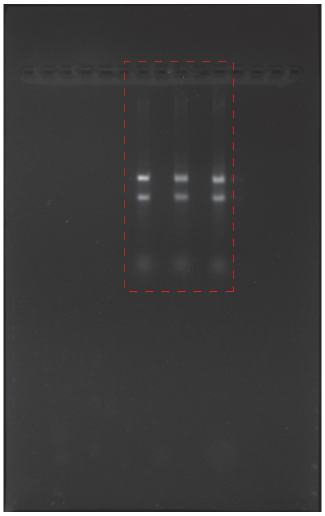

Total RNA

Extended Data Fig. 6a

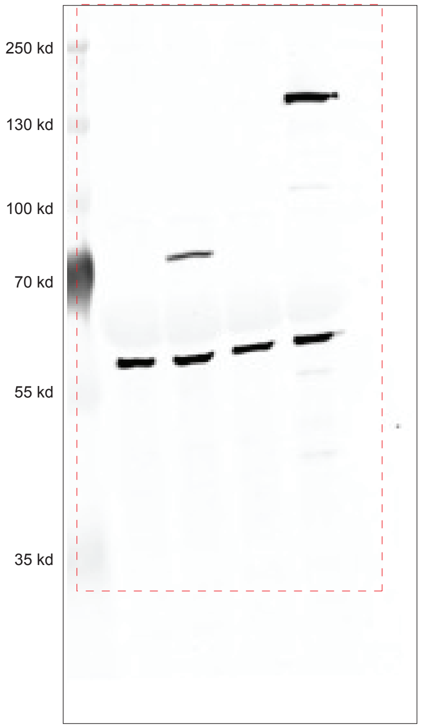

Anti-His

Extended Data Fig. 8b

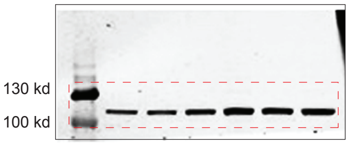

Anti-RpoB

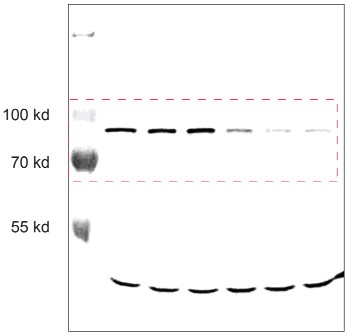

Anti-Rho

Supplementary Table Legends

Supplementary Table 1. TSS information for Mtb and Msm detected by SEnd-seq
For each TSS, its genomic position, direction, and average intensity are shown. Mtb TSSs are further categorized based on their location and orientation, and the leaderless TSSs are included in the gTSS group. Mtb TSSs without another strong TSS within 700 nt downstream are shown on a separate sheet.

Supplementary Table 2. TTS Information for Mtb and Msm detected by SEnd-seq
For each TTS, its genomic position, direction, and average intensity are shown.

Supplementary Table 3. Antisense transcript information for Mtb detected by SEnd-seq
For each antisense transcript, its start and end positions, end location category, length, and the corresponding coding transcription unit information are shown.

Supplementary Table 4. Transcription unit (TU) information annotated for the Mtb transcriptome
For each coding TU, its start and end positions, direction, and genes included (count, names) are shown. The TUs with a length over 700 nt are marked as "selected" in column J for further analysis.

Supplementary Table 5. Oligonucleotides and plasmids used in this study
All oligonucleotides used in this study were synthesized by Integrated DNA Technologies.
