# Supplementary material for: Incomplete transcripts dominate the Mycobacterium tuberculosis transcriptome
Source: Nature. 2024 Feb 28;627(8003):424–30. doi: 10.1038/s41586-024-07105-9 (PMC10937400; doi:10.1038/s41586-024-07105-9)
Supplement: Supplementary file 2 — Reporting Summary [file 41586_2024_7105_MOESM2_ESM.pdf]

nature portfolio

Corresponding author(s): Shixin Liu, Jeremy Rock

Last updated by author(s): 12/21/2023

Reporting Summary

Nature Portfolio wishes to improve the reproducibility of the work that we publish. This form provides structure for consistency and transparency in reporting. For further information on Nature Portfolio policies, see our Editorial Policies and the Editorial Policy Checklist.

Statistics

For all statistical analyses, confirm that the following items are present in the figure legend, table legend, main text, or Methods section.

n/a	Confirmed	
☐	☒	The exact sample size (*n*) for each experimental group/condition, given as a discrete number and unit of measurement
☐	☒	A statement on whether measurements were taken from distinct samples or whether the same sample was measured repeatedly
☐	☒	The statistical test(s) used AND whether they are one- or two-sided<br>*Only common tests should be described solely by name; describe more complex techniques in the Methods section.*
☒	☐	A description of all covariates tested
☒	☐	A description of any assumptions or corrections, such as tests of normality and adjustment for multiple comparisons
☐	☒	A full description of the statistical parameters including central tendency (e.g. means) or other basic estimates (e.g. regression coefficient) AND variation (e.g. standard deviation) or associated estimates of uncertainty (e.g. confidence intervals)
☐	☒	For null hypothesis testing, the test statistic (e.g. *F*, *t*, *r*) with confidence intervals, effect sizes, degrees of freedom and *P* value noted<br>*Give P values as exact values whenever suitable.*
☒	☐	For Bayesian analysis, information on the choice of priors and Markov chain Monte Carlo settings
☒	☐	For hierarchical and complex designs, identification of the appropriate level for tests and full reporting of outcomes
☐	☒	Estimates of effect sizes (e.g. Cohen's *d*, Pearson's *r*), indicating how they were calculated

Our web collection on statistics for biologists contains articles on many of the points above.

Software and code

Policy information about availability of computer code

Data collection	The sequencing data were collected on Illumina platforms, including NextSeq 500, NovaSeq 6000, at The Rockefeller University Genomics Resource Center.
Data analysis	After quality filtering and Illumina sequencing adaptor trimming with FASTX-Toolkit (v0.0.13) , the raw paired-end reads were merged to single-end reads by using FLASh software (v1.2.11, T. Magoc et al., 2011). The correlated 5'-end and 3'-end sequences were extracted by the custom script ('fasta_to_paired.sh') utilizing the SeqKit (v2.4.0) and Cutadapt (v4.1) packages. The inferred full-length reads were generated by Bedtools (v2.31.0, Quinlan et al., 2010) and Samtools (v1.17, Danecek et al., 2021) after mapping to the reference genome (NC000913.3 for Eco, NC008596.1 for Msm, and NC018143.2 for Mtb) via Bowtie2 (v2.5.1, Langmead et al., 2012). The full-length reads with an insert length greater than 10,000 nt were discarded. The mapping results were visualized using the IGV genome viewer (v2.4.10, Robinson et al., 2011). SEnd-seq coverage was calculated per nucleotide position in the genome, and further analyses were performed using Perl (v5.34.1) or Python (3.11.3) with custom scripts available on Github (https://github.com/LiuLab-codes/Mtb_transcriptome_profiling). Data analysis and visualization scripts utilized Python packages including Matplotlib (v3.7.1), Numpy (v1.24.3), Scipy (v1.10.1), bioinfokit (v0.3), and pyCircos (v0.3.0). Motif analysis was conducted using the MEME suite (v5.5.2, Bailey et al., 2015). The Gene Ontology analysis was performed on DAVID website (v2023q2, Sherman et al., 2022).

For manuscripts utilizing custom algorithms or software that are central to the research but not yet described in published literature, software must be made available to editors and reviewers. We strongly encourage code deposition in a community repository (e.g. GitHub). See the Nature Portfolio guidelines for submitting code & software for further information.

Data

Policy information about availability of data

All manuscripts must include a data availability statement. This statement should provide the following information, where applicable:

- Accession codes, unique identifiers, or web links for publicly available datasets
- A description of any restrictions on data availability
- For clinical datasets or third party data, please ensure that the statement adheres to our policy

SEnd-seq and ChIP-seq datasets from this study are deposited in Gene Expression Omnibus (GEO) with the accession number GSE211992 (BioProject PRJNA873109).

Human research participants

Policy information about studies involving human research participants and Sex and Gender in Research.

Reporting on sex and gender	N/A
Population characteristics	N/A
Recruitment	N/A
Ethics oversight	N/A

Note that full information on the approval of the study protocol must also be provided in the manuscript.

Field-specific reporting

Please select the one below that is the best fit for your research. If you are not sure, read the appropriate sections before making your selection.

☒ Life sciences ☐ Behavioural & social sciences ☐ Ecological, evolutionary & environmental sciences

For a reference copy of the document with all sections, see nature.com/documents/nr-reporting-summary-flat.pdf

Life sciences study design

All studies must disclose on these points even when the disclosure is negative.

Sample size	Sequencing experiments were conducted at least twice for each condition, and all other experiments were repeated at least three times, unless specified otherwise. For RNA samples, we collected from 4 mL of log-phase cells, and for ChIP-seq, samples were taken from 50 mL of log-phase cells. The SEnd-seq sample was sequenced to a depth more than 8 million reads, and the ChIP-seq sample to a depth of 3-10 million reads. These depths are sufficient for a comprehensive characterization of the transcriptome and ChIP signals in each sample, aligning with standard practices in the field.
Data exclusions	Low-quality reads and any paired-end sequences with an alignment insert length exceeding 10,000 nucleotides were excluded from subsequent data analysis. Other specific criteria are described in figure legends.
Replication	Sequencing experiments were conducted at least twice for each condition, and all other experiments were repeated at least three times, unless otherwise specified. 3 technical replicates were performed for qPCR measurements.
Randomization	This study didn’t include experiments that required randomization, as there was no allocation of samples into groups.
Blinding	This study didn't include experiments that required blinding, as all experiments were quantitative and there was no allocation of samples into groups.

Reporting for specific materials, systems and methods

We require information from authors about some types of materials, experimental systems and methods used in many studies. Here, indicate whether each material, system or method listed is relevant to your study. If you are not sure if a list item applies to your research, read the appropriate section before selecting a response.

Materials & experimental systems

n/a	Involved in the study
☐	☒ Antibodies
☒	☐ Eukaryotic cell lines
☒	☐ Palaeontology and archaeology
☒	☐ Animals and other organisms
☒	☐ Clinical data
☒	☐ Dual use research of concern

Methods

n/a	Involved in the study
☐	☒ ChIP-seq
☒	☐ Flow cytometry
☒	☐ MRI-based neuroimaging

Antibodies

Antibodies used

Antibodies against Mtb Rho (a gift from D. Schnappinger, Weill Cornell Medicine), against Eco RpoB (BioLegend, 663903, developed with a peptide fragment common to bacterial RpoB), against Eco sigma 70 (BioLegend, 663208, developed with a peptide fragment of the common bacterial housekeeping sigma factor) and against His-Tag (Santa cruze, sc-8036) were used.

Validation

The antibody against Mtb Rho was previously validated by western blot (PMID:28348398). The antibody against Eco RpoB was previously used in Mtb RpoB ChIP-seq experiments (PMID: 23222129) and validated by western blot (PMID: 30242166). The antibody against Eco Sigma70 was previously used in Msm SigA ChIP-seq experiments (PMID: 25089258). The antibody against His-Tag was previously used in western blot experiments (PMID: 37154023).

ChIP-seq

Data deposition

☒ Confirm that both raw and final processed data have been deposited in a public database such as GEO.

☒ Confirm that you have deposited or provided access to graph files (e.g. BED files) for the called peaks.

Data access links
May remain private before publication.

SEnd-seq, NET-SEnd-seq, and ChIP-seq datasets from this study are deposited in Gene Expression Omnibus (GEO) with the accession number GSE211992 (BioProject PRJNA873109).

Files in database submission

ChIP_seq_WT_input_rep1_R1_001.fastq.gz ChIP_seq_WT_input_rep1_R2_001.fastq.gz
ChIP_seq_WT_input_rep2_R1_001.fastq.gz ChIP_seq_WT_input_rep2_R2_001.fastq.gz
ChIP_seq_WT_Ab_RpoB_rep1_R1_001.fastq.gz ChIP_seq_WT_Ab_RpoB_rep1_R2_001.fastq.gz
ChIP_seq_WT_Ab_RpoB_rep2_R1_001.fastq.gz ChIP_seq_WT_Ab_RpoB_rep2_R2_001.fastq.gz
ChIP_seq_WT_DMSO_input_rep1_R1_001.fastq.gz ChIP_seq_WT_DMSO_input_rep1_R2_001.fastq.gz
ChIP_seq_WT_RIF_input_rep1_R1_001.fastq.gz ChIP_seq_WT_RIF_input_rep1_R2_001.fastq.gz
ChIP_seq_WT_DMSO_Ab_RpoB_rep1_R1_001.fastq.gz ChIP_seq_WT_DMSO_Ab_RpoB_rep1_R2_001.fastq.gz
ChIP_seq_WT_DMSO_Ab_RpoB_rep2_R1_001.fastq.gz ChIP_seq_WT_DMSO_Ab_RpoB_rep2_R2_001.fastq.gz
ChIP_seq_WT_RIF_Ab_RpoB_rep1_R1_001.fastq.gz ChIP_seq_WT_RIF_Ab_RpoB_rep1_R2_001.fastq.gz
ChIP_seq_WT_RIF_Ab_RpoB_rep2_R1_001.fastq.gz ChIP_seq_WT_RIF_Ab_RpoB_rep2_R2_001.fastq.gz
ChIP_seq_WT_Ab_SigA_rep1_R1_001.fastq.gz ChIP_seq_WT_Ab_SigA_rep1_R2_001.fastq.gz
ChIP_seq_WT_Ab_SigA_rep2_R1_001.fastq.gz ChIP_seq_WT_Ab_SigA_rep2_R2_001.fastq.gz
ChIP_seq_WT_Ab_SigA_rep3_R1_001.fastq.gz ChIP_seq_WT_Ab_SigA_rep3_R2_001.fastq.gz

Genome browser session
(e.g. UCSC)

No longer applicable.

Methodology

Replicates

At least two replicates were prepared for each sample.

Sequencing depth

WT_input_rep1, pair-end, read number: 1648782, read-length: 75 bp
WT_input_rep2, pair-end, read number: 1447153, read-length: 75 bp
ChIP_seq_WT_Ab_RpoB_rep1, pair-end, read number:10254546, read-length: 150 bp
ChIP_seq_WT_Ab_RpoB_rep2, pair-end, read number:10799500, read-length: 150 bp
ChIP_seq_WT_Ab_SigA_rep1, pair-end, read number: 4971901, read-length: 150 bp
ChIP_seq_WT_Ab_SigA_rep2, pair-end, read number: 5360395, read-length: 150 bp
ChIP_seq_WT_Ab_SigA_rep3, pair-end, read number: 4896344, read-length: 150 bp
WT_DMSO_input, pair-end, read number: 3740538, read-length: 75 bp
WT_RIF_input, pair-end, read number: 3445471, read-length: 75 bp
ChIP_seq_WT_DMSO_Ab_RpoB_rep1, pair-end, read number: 7397067, read-length: 75 bp
ChIP_seq_WT_DMSO_Ab_RpoB_rep2, pair-end, read number: 7931837, read-length: 75 bp
ChIP_seq_WT_RIF_Ab_RpoB_rep1, pair-end, read number:7954605, read-length: 75 bp
ChIP_seq_WT_RIF_Ab_RpoB_rep2, pair-end, read number:6939423, read-length: 75 bp

Antibodies

The antibody against Eco RpoB (BioLegend, 663903) was used in the Mtb RpoB ChIP-seq experiments. The antibody against Eco Sigma70 (BioLegend, 663208) was used in the Mtb SigA ChIP-seq experiments.

Peak calling parameters	We analyzed the signal coverage with custom scripts.
Data quality	Reads with high quality (quality score >=30) were retained using FASTX-Toolkit (v0.0.13) and only uniquely mapped reads were used for downstream analysis.
Software	Samtools (version 1.18), Bowtie2 (version 2.5.1), Bedtools (version 2.31.0), Python (version 3.11.3), Matplotlib (v3.7.1), Numpy (v1.24.3), and custom scripts were used.
